# Estimating the Global and Regional Burden of Streptococcus pneumoniae Meningitis in Children: Protocol for a Systematic Review and Meta-Analysis

**DOI:** 10.2196/50678

**Published:** 2024-07-16

**Authors:** Jay J Park, Jakov Tiefenbach, Mohammed Ma'arij Anwar, Sandhya Narayanan, Beatrice Ope, Selene Seo Bin Han, Boni Maxime Ale, Davies Adeloye, Igor Rudan

**Affiliations:** 1 Edinburgh Medical School University of Edinburgh Edinburgh United Kingdom; 2 Edinburgh Global Health Interest Group Edinburgh United Kingdom; 3 Neurological Institute Cleveland Clinic Cleveland, OH United States; 4 Department of Bioengineering Imperial College London London United Kingdom; 5 Barnardo’s Barkingside United Kingdom; 6 Association for Reproductive and Family Health Abuja Nigeria; 7 Holo Healthcare Limited Nairobi Kenya; 8 School of Health and Life Sciences Teesside University Middlesbrough United Kingdom; 9 Centre of Global Health, Edinburgh Medical School Usher Institute University of Edinburgh Edinburgh United Kingdom

**Keywords:** Streptococcus pneumoniae, meningitis, streptococcal meningitis, pneumococcal meningits, global burden, pediatric meningitis, infectious disease, pneumonia, sepsis, infection, infection prevention and control, IPC, child health, global health, systematic review, pneumococcal

## Abstract

**Background:**

*Streptococcus pneumoniae* (Spn) has been a leading cause of bacterial meningitis in children. The most recent estimation of the global burden of Spn meningitis indicates a positive trajectory in eliminating Spn through the implementation of pneumococcal conjugate vaccines. However, continuous monitoring and assessment of the disease burden are necessary due to the evidence of serotype replacement, antibiotic resistance, and the impact of the recent COVID-19 pandemic.

**Objective:**

The aim of this systematic review is to provide an updated and focused assessment of the global and regional burden of Spn meningitis in children, which can guide policies and strategies to reduce the disease burden.

**Methods:**

Population-based studies published from January 1, 2000, to January 1, 2022, were preliminarily searched from the electronic databases PubMed, Embase, Global Health (CABI), and CINAHL Plus without any language restrictions. Studies were included if they reported the incidence, prevalence, mortality, or case-fatality ratio (CFR) for Spn meningitis in children aged 0-4 years; meningitis was confirmed by cerebrospinal fluid culture; the study period was a minimum of 1 year; the number of reported cases was at least 10; and the study had no methodological ambiguities. The article screening process follows the PRISMA (Preferred Reporting Items for Systematic reviews and Meta-Analyses) guidelines. Characteristics including study period, setting, World Health Organization region, income level, vaccination information, and participant data (age, number of cases, deaths, sequelae, and risk factors) will be extracted from the included studies. Search results will be updated and incorporated into our review prior to finalizing the extraction of data. Generalized linear mixed models meta-analysis will be performed to estimate the pooled incidence and CFR. We will further assess the risk of bias and heterogeneity, and will perform subgroup and sensitivity analyses to provide a meaningful interpretation of the current burden and literature for pneumococcal meningitis.

**Results:**

Our preliminary search in December 2021 yielded 9295 articles. Out of 275 studies that were assessed with our eligibility criteria, 117 articles were included. Data extraction and analysis are expected to be complete by January 2025. We plan to publish the results from the full study, including an updated search in 2024, by March 2025.

**Conclusions:**

Given that the major burden of Spn meningitis affects children under the age of 5 years, this systematic review will provide a thorough understanding of the global burden of Spn meningitis in this vulnerable population over a span of 2 decades. Insights into incidence trends, geospatial distribution, risk factors, and sequelae will be valuable for stakeholders, policy makers, and the academic community. This information will aid in the ongoing monitoring of the disease and in enhancing targeted vaccine programs to further mitigate the impact of the disease on children worldwide.

**Trial Registration:**

PROSPERO CRD42021293110; https://tinyurl.com/kc3j5k4m

**International Registered Report Identifier (IRRID):**

DERR1-10.2196/50678

## Introduction

*Streptococcus pneumoniae* (Spn) is a gram-positive, encapsulated coccus; the World Health Organization (WHO) has identified diseases caused by Spn as a serious public health concern [1]. Indeed, Spn infection is a major cause of morbidity and mortality worldwide and is a leading cause of bacterial meningitis in children [2]. There were an estimated 103,000 cases of Spn globally with a case-fatality ratio (CFR) of 59% in 2000 [3]. With the introduction of pneumococcal conjugate vaccines (PCVs), the most recent study into the global burden of Spn in 2015 reported a decrease in burden to 83,900 cases (range 36,100-169,000) of pneumococcal meningitis with a CFR of 44% in children regardless of HIV status [4].

In 2007, the WHO recommended including the 7-valent polysaccharide-protein conjugate vaccine (PCV7) in national immunization programs, particularly in countries with high mortality rates from pneumococcal disease in children under 5 years [1]. PCV7 has been widely used for providing protection against 65%-85% of serotypes associated with invasive pneumococcal disease in young children [1]. However, since 2009, the WHO has recommended PCV10 or PCV13 depending on the country’s serotype prevalence, financial budget, and vaccine supply; thus, PCV7 is no longer in use [5]. Multiple notable trials worldwide have been conducted and are currently ongoing [6-11] to provide evidence for the preferred product and dosage schedule, which is currently recommended to be 3+0 or 2+1 [12]. As of December 2021, 147 countries (114 with PCV13, 26 with PCV10, and 7 with PCV10&PCV13) have implemented PCV into their national immunization programs, 60 of which are financially supported by the Global Alliance for Vaccines and Immunization [13]. The universalization of PCV has been estimated to have prevented 250,000 deaths in children from 2000 to 2015 [4].

Despite such continuous endeavors to globally implement PCV, the emergence of COVID-19 and associated pandemic measures affected a shift in the paradigm of Spn meningitis. The mass distancing measures contributed to a sharp decline in pneumococcal infections at the start of the pandemic [14]; however, disruption of vaccination programs has become prevalent and concerning [15,16]. Evidence forecasts that this phenomenon will lead to a detrimental increase in the cases of Spn meningitis [17]. The true impact of COVID-19 on the current Spn disease burden is yet to be determined. Therefore, an updated estimation of Spn meningitis will be an important comparator for future investigations and guide strategies to mitigate the pandemic’s negative impacts on Spn immunization programs.

Although the introduction of the vaccine has contributed to a decreasing trend in the burden of pneumococcal meningitis, the reported figures show a marginal reduction within the 15-year period [3,4]. Recent evidence highlights a concerning outlook for the future burden of the disease. Although vaccination has reduced the incidence of Spn for PCV serotypes, there has not been a uniform decrease in the incidence and CFR of Spn meningitis, revealing an issue of serotype replacement [18-21]. This predicament is exacerbated by the increasing antibiotic resistance to Spn meningitis [20,22,23]. Heterogeneity of serotypes and antibiotic resistance are persistent challenges facing other causes of bacterial meningitis, which demands the Spn burden to be scrutinized to a similar degree [24]. Ultimately, Wahl et al [4] reported that their study did not account for the outbreaks of pneumococcal meningitis, possibly leading to an underestimation. Furthermore, the current literature concurs with these authors’ concern about the countries that are yet to implement a national pneumococcal immunization program [4,14]. These problems call for further rigorous efforts in determining and monitoring the disease burden to direct policies to counter the numerous cases and deaths [4].

This review aims to provide the most up-to-date estimate of the global and regional burden of Spn meningitis in children under the age of 5 years. Our objective is to provide meaningful data regarding the global and regional incidence, CFR, and risk factors to guide policies in preventing and minimizing the burden of the disease.

## Methods

### Study Design and Registration

A systematic review of the literature will be performed to estimate and understand the global and regional burden of Spn meningitis in children under the age of 5 years. This protocol was developed in accordance with the PRISMA-P (Preferred Reporting Items for Systematic reviews and Meta-Analyses Protocols) checklist [25]. The review was registered with the International Prospective Register of Systematic Reviews (PROSPERO) on December 3, 2021. Any amendments to the protocol will be subsequently published on the register (CRD42021293110).

### Inclusion Criteria

This review will include studies published between January 1, 2000, and January 1, 2024, based on a search of the following databases: PubMed, Embase, Global Health (CABI), and CINAHL Plus. The initial search was conducted for literature published from January 1, 2000, to December 1, 2021, and the search will be reconducted to include the most recent papers prior to final data extraction and analysis. Meningitis cases that we will include in the review will be defined as an acute bacterial illness with symptoms consisting of severe headache, nausea, neck stiffness, vomiting, photophobia, and high fever. Clinical presentations denoted in the studies will be compared to the definitions from the *International Classification of Diseases, Tenth Revision* [26] and WHO [27-29].

Population-based studies will be included if they (1) reported the incidence, prevalence, mortality, or CFR for pneumococcal meningitis (the papers have to specify or have clear suggestions that *Streptococcus* was referring to Spn); (2) confirmed meningitis by cerebrospinal fluid culture in a facility-based laboratory; (3) had a minimum study period of 1 year (or multiples of 1 year); (4) reported a minimum of 10 cases of confirmed Spn meningitis during the study period; (5) reported results for a participant age group of 0-4 years; and (6) contained no methodological ambiguities about how the study was conducted and no systematic errors in the design in the research that could affect the results of the study.

### Search Strategy

We plan to use the following search strategy to capture all relevant studies without any restrictions for the language of publication: Meningitis AND (*Streptococcus pneumoniae* OR invasive pneumococcal disease OR streptococcal OR pneumococcal) AND (burden OR incidence OR prevalence OR deaths OR mortality OR morbidity OR sequela* OR case fatality OR risk factor OR vaccination OR immunisation) NOT (case reports OR animals OR adults). This search was performed on December 3, 2021. The search strategies used in each database are provided in Multimedia Appendix 1.

### Study Records

The screening process will be performed on EndNote20 (Clarivate Analytics). Two reviewers (JT and MMA) will independently screen the studies for titles and abstracts that are relevant to this review. Subsequently, the reviewers will only discuss their screening results when applying eligibility criteria for the retrieved full texts. A third reviewer (JJP) will check for final inclusions and settle any disagreements when applying the eligibility criteria. Any decisions that are outstanding will be discussed with a fourth reviewer (DA).

### Data Items

Our data extraction proforma (Table 1) has been developed to fully answer our review question according to the condition, context, and population (CoCoPop) framework [30]. This proforma has been drafted according to a pilot form that has been completed by two independent reviewers (JJP and JT). Once screened for all studies that are relevant, data extraction for the systematic review will be performed by three authors (JT, SN, and MMA) and verified by a fourth author (JJP). Any disagreements will be settled by these four authors through consensus-based discussion, and subsequent unsettled decisions will be discussed with the fifth author (DA). All data extracted from included studies will be managed and stored in an Excel spreadsheet.

The items in Table 1 will be extracted from all included studies. Missing data that are unavailable in the retrieved full texts will be acquired by contacting the authors of the studies. If unreported details remain, we will report the missing data. Case ascertainment of pneumococcal meningitis will have to be available for the study to be included in our review.

**Table 1:** Data extraction proforma to outline and define specific data to be extracted from included studies.

Data to be extracted		Definitions
**Study characteristics**		
	Authors	Authors of the study
	Date of publication	Year in which the study is published
	Study period	Year(s) in which the study was conducted
	Study era	Whether the study was conducted before or after 2010
	Study setting	Community-based study versus hospital-based study
	Study site	Country in which the study was conducted (and city if defined in study)
	WHO^a^ region of the study	WHO classification of the region in which the study is conducted [31]
	Income level of the study site	World Bank classification of the income level of the study site [32]
	Study design	Retrospective versus prospective studies
	Study duration	Duration of the study in months
	Study quality	Quality of the study (ie, high, moderate, low, very low)
**Vaccination**		
	Vaccination era	Pre- versus postvaccination implementation
	Vaccine introduction year	Year in which vaccination program has been implemented
	Vaccination program	Type of vaccination program at the time of study (ie, PCV^b^7, PCV10, PCV13, PCV10&13)
	Dosage	Dosage plan of the vaccination at the time of study (ie, 3+0, 2+1, or 3+1)
**Participants**		
	Diagnostic criteria	How the diagnosis is made (ie, latex agglutination, cell count, polymerase chain reaction, isolation from the cerebrospinal fluid, isolation from the blood serology, enzyme-linked immunosorbent assay)
	Age range	Age range of the participants in months
	Number of cases confirmed	The number of pneumococcal meningitis cases confirmed within the study
	Number of deaths confirmed	Number of deaths due to pneumococcal meningitis confirmed within the study
	Population denominator	Total samples in the study; catchment population of the surveillance
	Sequelae	The long-term consequences due to Spn^c^ meningitis
	Number of patients with sequelae	Number of patients that experienced specific long-term consequence due to Spn meningitis
**Risk factors**		
	Risk group	Type of risk identified in the study (eg, age, sex, hospital-acquired, comorbidities)
	Risk reference (comparator)	Variable that the risk is being compared to
	Risk	Risk factors identified within the study
	Measure of effect	Measure of effect for the risk factor (eg, odds ratio, relative risk)
	Upper confidence interval	Maximum value of the confidence interval
	Lower confidence interval	Minimum value of the confidence interval

^a^WHO: World Health Organization.

^b^PCV: pneumococcal conjugate vaccine.

^c^Spn: *Streptococcus pneumoniae.*

### Outcomes and Prioritization

The primary outcome of this review will be the global and regional incidence and CFR of pneumococcal meningitis in children. Our secondary outcomes include exploring the different subgroups and their impact on the incidence and CFR. In addition, if sufficient data for risk factors are identified from the included studies (10 or more), our objective is to produce meaningful multivariable risk factors of Spn meningitis.

### Risk of Bias

The quality of the included studies will be appraised by two independent reviewers (JJP and SN). Any disagreements regarding the quality of the study will be discussed among four reviewers (JJP, SN, JT, and MMA). If necessary, an opinion will be sought from a fifth reviewer (DA).

A consensus on a quality assessment tool for observational studies, and especially incidence/prevalence studies, is yet to be established [33,34]. Therefore, we have improved upon our own quality assessment criteria that have been adapted from the validated checklists/quality assessment tools from the Joanna Briggs Institute Critical Appraisal Tools; Checklist for Prevalence Studies; National Heart, Lung, and Blood Institute Quality Assessment Tool for Observational Cohort and Cross-Sectional Studies; and GRADE (Grading of Recommendations Assessment, Development, and Evaluation). We will no longer be using a scoring system to assess the quality but rather a scale from “good” to “very low,” according to the recommendations in the literature [24,34]. This scale has been adapted so the questions are specific to the studies of inclusion (see Multimedia Appendix 2).

### Data Synthesis

The pooled incidence and CFR of Spn meningitis will be estimated by performing generalized linear mixed models meta-analysis in the case of determining mortality and the population denominator from all included studies. For our additional outcome, we will also construct a multivariable metaregression model to produce a pooled odds ratio for multivariable risk factors of Spn meningitis. Meta-analyses will be performed with the meta, metaphor, and dmetar packages of R statistical software (version 4.1.2).

Heterogeneity among studies will be assessed using *I*^2^ statistics, and heterogeneity will be further explored by performing subgroup analysis on the following extracted items: WHO region, income level of the study site, study period, and vaccination period.

We will be performing the Egger regression test to assess for publication bias. A funnel plot of standard error by incidence will be produced and visually inspected for asymmetry. Sensitivity analysis will be performed to assess the impact of the study design, study setting, and quality of the studies on the incidence/CFR.

### Ethical Considerations

Formal ethical approval will not be required as this study will not involve primary data collection or patients.

## Results

**Figure 1:**
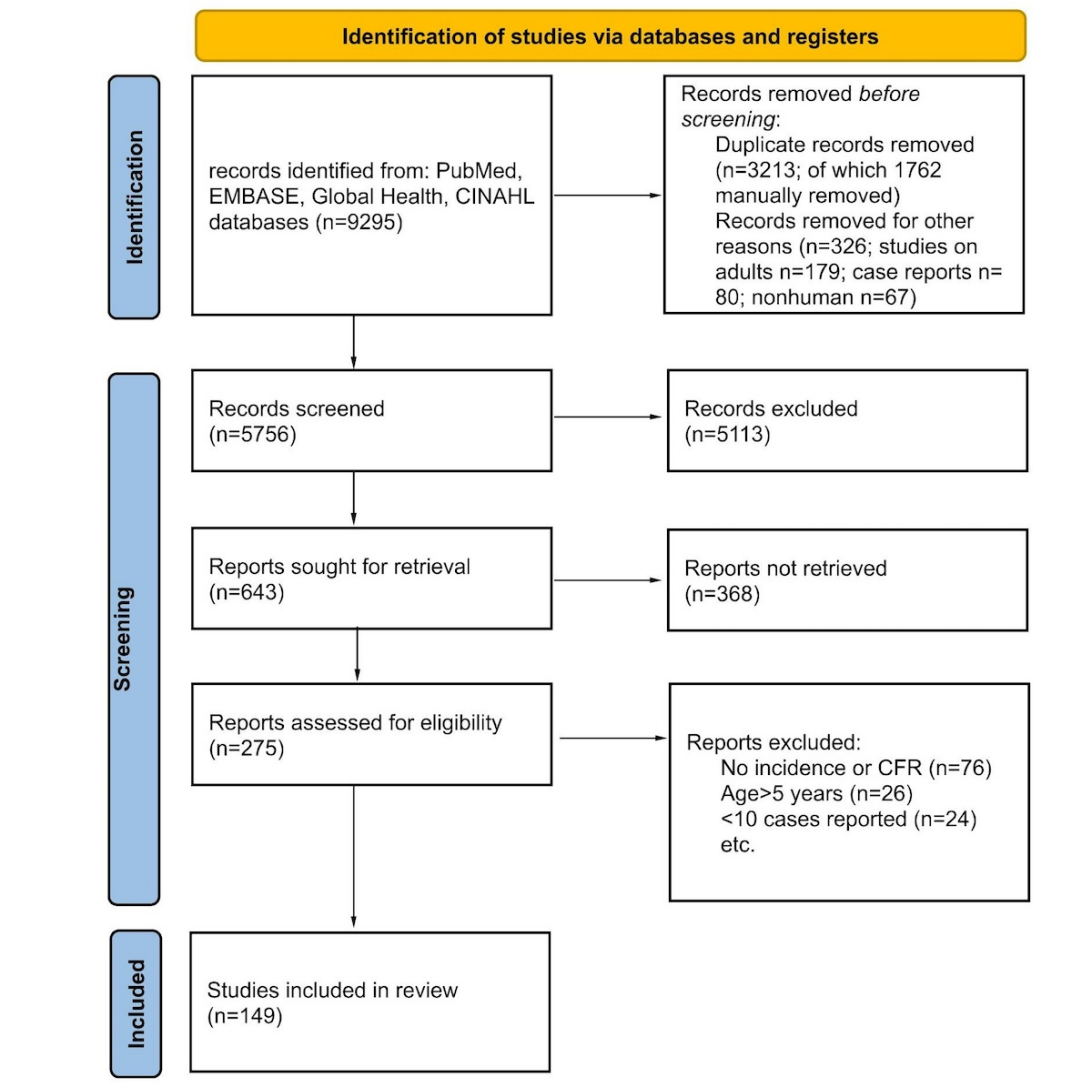
PRISMA (Preferred Reporting Items for Systematic reviews and Meta-Analyses) flowchart to demonstrate the screening process for studies included in this systematic review. CFR: case fatality ratio.

The search from 4 databases resulted in 9295 articles, as shown in the PRISMA flowchart in Figure 1.

A total of 3213 articles were removed as duplicates and 326 studies were further removed due to the participants being adults or nonhuman or the articles being case reports. After the screening process, we included a total of 117 articles that met our eligibility criteria. We are currently undergoing the data extraction process, which will soon be followed by a meta-analysis. The expected completion date for extraction and analysis is January 2025 and the results are planned to be published by March 2025. The results of the completed study will be disseminated through international conferences and publication in a peer-reviewed journal.

## Discussion

This systematic review will identify the most recent global burden of Spn meningitis in children under 5 years of age, while providing important insights into factors that may contribute to a greater or lesser disease burden. Moreover, considering the frequency of sequelae following Spn meningitis, our study will comprehensively capture the types and incidence of the long-term clinical consequences associated with Spn meningitis in children [35].

Findings from our previous systematic review on *Haemophilus influenzae* type B (Hib) meningitis concurred with a long-standing challenge in the epidemiological literature [36,37], which is the scarcity of surveillance data in countries where such data are most needed. The least amount of data were acquired from the Southeast Asia Region and Eastern Mediterranean Region, which may have impacted the accuracy of our estimation. Therefore, this review will aim to assess and highlight the gaps in the evidence of Spn meningitis in children. For the same reason, we will include an assessment of outcomes and the body of evidence through the GRADE approach, using the GRADEpro Guideline Development Tool. Two independent reviewers (JJP and SN) will assess the quality of the outcomes. The third reviewer will adjudicate any disagreements that are unsettled between the two reviewers.

Although recent evidence indicates the decrease in language bias due to the trend of studies being published in English [37], our review on Hib meningitis had 6 studies that were published in a non-English language out of the 33 studies that were included. As a result, one of the major strengths of this review is including studies of all languages. We will be using Google Translate to acquire data from non-English studies and, similar to our previous review, we will seek advice from external individuals who speak the corresponding languages as necessary. This is to improve the accuracy of extracting data by understanding subtle nuances within the publication.

There are, however, some limitations to this review. We will not be including unpublished data from registers such as the WHO Invasive Bacterial Disease Surveillance Network, which may lead to an underestimation of the disease burden. In addition, we will focus on providing a crude estimate, which may be different from the adjusted values when factors such as HIV prevalence and vaccination have been considered. Nevertheless, this systematic review and meta-analysis will provide data that are comparable to the burden reported in the previous literature and will further expand our knowledge of the current global disease burden of Spn meningitis in children and the impact of universal vaccination programs.

With a specific focus on Spn meningitis in the pediatric population, this review is positioned to offer deeper insights into the disease burden and provide valuable guidance for managing Spn at a policy level for this vulnerable patient group.

## Appendix

Multimedia Appendix 1 Search terms and strategy.

Multimedia Appendix 2 Quality assessment criteria for assessing risk of bias.

Multimedia Appendix 3 Preferred Reporting Items for Systematic Review and Meta-Analysis Protocols (PRISMA-P) checklist.

## Abbreviations

CFR: case-fatality-ratio
CoCoPop: condition, context, and population
GRADE: Grading of Recommendations Assessment, Development, and Evaluation
Hib: Haemophilus influenzae type B
PCV: pneumococcal conjugate vaccines
PRISMA-P: Preferred Reporting Items for Systematic Reviews and Meta-Analyses Protocols
PROSPERO: Prospective Register of Systematic Reviews
Spn: *Streptococcus pneumoniae*
WHO: World Health Organization
