# Supplementary material for: Estimating the Global and Regional Burden of Streptococcus pneumoniae Meningitis in Children: Protocol for a Systematic Review and Meta-Analysis
Source: JMIR Res Protoc. 2024 Jul 16;13:e50678. doi: 10.2196/50678 (PMC11289570; doi:10.2196/50678)
Supplement: Multimedia Appendix 1 [file resprot_v13i1e50678_app1.pdf]

Search terms

PubMed

Number	Search Terms	Results
1	((("meningeal"[All Fields] OR "meninges"[MeSH Terms] OR "meninges"[All Fields] OR "meninge"[All Fields] OR "meningism"[MeSH Terms] OR "meningism"[All Fields] OR "meningisms"[All Fields] OR "meningitis"[MeSH Terms] OR "meningitis"[All Fields] OR "meningitides"[All Fields]) AND ("streptococcus pneumoniae"[MeSH Terms] OR ("streptococcus"[All Fields] AND "pneumoniae"[All Fields]) OR "streptococcus pneumoniae"[All Fields] OR (("invasibility"[All Fields] OR "invasible"[All Fields] OR "invasion"[All Fields] OR "invasions"[All Fields] OR "invasive"[All Fields] OR "invasively"[All Fields] OR "invasiveness"[All Fields] OR "invasives"[All Fields] OR "invasivity"[All Fields]) AND ("pneumococcal infections"[MeSH Terms] OR ("pneumococcal"[All Fields] AND "infections"[All Fields]) OR "pneumococcal infections"[All Fields] OR ("pneumococcal"[All Fields] AND "disease"[All Fields]) OR "pneumococcal disease"[All Fields])) OR ("streptococcus"[MeSH Terms] OR "streptococcus"[All Fields] OR "streptococcal"[All Fields]) OR ("pneumococcal vaccines"[MeSH Terms] OR ("pneumococcal"[All Fields] AND "vaccines"[All Fields]) OR "pneumococcal vaccines"[All Fields] OR "pneumococcal"[All Fields])) AND ("burden"[All Fields] OR "burdened"[All Fields] OR "burdening"[All Fields] OR "burdens"[All Fields] OR ("epidemiology"[MeSH Subheading] OR "epidemiology"[All Fields] OR "incidence"[All Fields] OR "incidence"[MeSH Terms] OR "incidences"[All Fields] OR "incident"[All Fields] OR "incidents"[All Fields]) OR ("epidemiology"[MeSH Subheading] OR "epidemiology"[All Fields] OR "prevalence"[All Fields] OR "prevalence"[MeSH Terms] OR "prevalance"[All Fields] OR "prevalences"[All Fields] OR "prevalence s"[All Fields] OR "prevalent"[All Fields] OR "prevalently"[All Fields] OR "prevalents"[All Fields]) OR ("death"[MeSH Terms] OR "death"[All Fields] OR "deaths"[All Fields]) OR ("mortality"[MeSH Terms] OR "mortality"[All Fields] OR "mortalities"[All Fields] OR "mortality"[MeSH Subheading]) OR ("epidemiology"[MeSH Subheading] OR "epidemiology"[All Fields] OR "morbidity"[All Fields] OR "morbidity"[MeSH Terms] OR "morbid"[All Fields] OR "morbidities"[All Fields] OR "morbids"[All Fields]) OR ("complications"[MeSH Subheading] OR "complications"[All Fields] OR "sequelae"[All Fields] OR "sequela"[All Fields] OR "sequelaes"[All Fields] OR "sequelas"[All Fields]) OR (("ieee int conf automation sci eng case"[Journal] OR "case phila"[Journal] OR "case"[All Fields]) AND ("fatal"[All Fields] OR "fatalities"[All Fields] OR "fatality"[All Fields] OR "fatally"[All Fields])) OR ("risk factors"[MeSH Terms] OR ("risk"[All Fields] AND "factors"[All Fields]) OR "risk factors"[All Fields] OR ("risk"[All Fields] AND "factor"[All Fields]) OR "risk factor"[All Fields]) OR ("vaccin"[Supplementary Concept] OR "vaccin"[All Fields] OR "vaccination"[MeSH Terms] OR "vaccination"[All Fields] OR "vaccinable"[All Fields] OR "vaccinal"[All Fields] OR "vaccinate"[All Fields] OR "vaccinated"[All Fields] OR "vaccinates"[All Fields] OR "vaccinating"[All Fields] OR "vaccinations"[All Fields] OR "vaccination s"[All Fields] OR "vaccinator"[All Fields] OR "vaccinators"[All Fields] OR "vaccine s"[All Fields] OR "vaccined"[All Fields] OR "vaccines"[MeSH Terms] OR "vaccines"[All Fields] OR "vaccine"[All	3270

Fields] OR "vaccins"[All Fields]) OR ("immune"[All Fields] OR "immuned"[All Fields] OR "immunes"[All Fields] OR "immunisation"[All Fields] OR "vaccination"[MeSH Terms] OR "vaccination"[All Fields] OR "immunization"[All Fields] OR "immunization"[MeSH Terms] OR "immunisations"[All Fields] OR "immunizations"[All Fields] OR "immunise"[All Fields] OR "immunised"[All Fields] OR "immuniser"[All Fields] OR "immunisers"[All Fields] OR "immunising"[All Fields] OR "immunities"[All Fields] OR "immunity"[MeSH Terms] OR "immunity"[All Fields] OR "immunization s"[All Fields] OR "immunize"[All Fields] OR "immunized"[All Fields] OR "immunizer"[All Fields] OR "immunizers"[All Fields] OR "immunizes"[All Fields] OR "immunizing"[All Fields]))) NOT ("case reports"[Publication Type] OR ("animals"[MeSH Terms:noexp] OR "animal"[All Fields]) OR "adult"[Title])) AND (2000:2021[pdat])

Embase

Number	Search Terms	Results
1	meningitis.af.	87878
2	(streptococcus pneumoniae or invasive pneumococcal disease or streptococcal or pneumococcal).af.	89534
3	(burden or incidence or prevalence or deaths or mortality or morbidity or sequela* or case fatality or risk factor or vaccin* or immunisation).af.	5417608
4	case report.pt. or adult*.ti. or animal*.ti.	575225
5	1 and 2 and 3	6556
6	5 not 4	6029
7	limit 6 to yr="2010 -Current"	3555

Global Health (CABI)

Number	Search Terms	Results

1	(((burden) OR (incidence) OR (prevalence) OR (deaths) OR (mortality) OR (morbidity) OR (sequela*) OR (case fatality) OR (risk factor) OR (vaccin*) OR (immuni*)) AND ((streptococcus pneumoniae) OR (invasive pneumococcal disease) OR (streptococcal) OR (pneumococcal)) AND ((meningitis))) NOT (it:(case report) OR title:(adult*) OR title:(animal*))<br>Refinements:<br>Year = 2021 OR 2020 OR 2019 OR 2018 OR 2017 OR 2016 OR 2015 OR 2014 OR 2013 OR 2012 OR 2011 OR 2010 OR 2008 OR 2009 OR 2007 OR 2006 OR 2000 OR 2001 OR 2003 OR 2005 OR 2004 OR 2002	1711

CINAHL Plus

Number	Search Terms	Results
S1	TX meningitis	8,473
S2	TX streptococcus pneumoniae OR TX invasive pneumococcal disease OR TX streptococcal OR TX pneumococcal	11,201
S3	TX burden OR TX incidence OR TX prevalence OR TX deaths OR TX mortality OR TX sequela* OR TX case fatality OR TX risk factor OR TX vaccin* OR TX immuni#ation	1,079,560
S4	PT case report OR TI adult* OR TI animal*	132,334
S5	S1 AND S2 AND S3	1,032
S6	S5 NOT S4<br>Limiters - Linked Full Text; Publication Year: 2000-2021	759
