# Supplementary material for: Estimating the Global and Regional Burden of Streptococcus pneumoniae Meningitis in Children: Protocol for a Systematic Review and Meta-Analysis
Source: JMIR Res Protoc. 2024 Jul 16;13:e50678. doi: 10.2196/50678 (PMC11289570; doi:10.2196/50678)
Supplement: Multimedia Appendix 2 [file resprot_v13i1e50678_app2.pdf]

Quality assessment criteria for assessing risk of bias.

Quality Criteria		Yes	No	Unclear	Not applicable
Study Design	Were the meningitis cases clearly defined: Clinical diagnosis and laboratory-confirmed diagnosis? (JBI, 6)*				
	Were lumbar puncture/CSF consistently implemented across ALL study participants? (JBI, 7)*				
	Were inclusion and/or exclusion criteria of participants prespecified and applied uniformly? (NHLBI,4)*				
Were the limitations stated in the study? (NHLBI, 14)*					
Sampling and Denominator	Was the population denominator clearly specified and defined? (JBI, 8)*				
	Were study participants recruited in an appropriate way? (JBI, 2)*				
	Was the sample representative of the target population? (JBI, 3)*				
**Criteria (High, Moderate, Low, Very Low)§**					

*() - indicates where the question has been adapted from.

§ - Quality criteria has been referenced from GRADE.
